# Predictive effects of platelet-to-lymphocyte ratio on neonatal thrombocytopenia in primary immune thrombocytopenic mothers: a retrospective cohort study

**DOI:** 10.1186/s12884-023-06010-9

**Published:** 2023-09-23

**Authors:** Huang Qionghui, Zeng Chaomei, Liu Jie, Qin Jiong

**Affiliations:** Department of Pediatrics, Peking University People’s Hospital, No.11 Xizhimen South Street, Xicheng District, Beijing, 100044 People’s Republic of China

**Keywords:** Blood platelets, Thrombocytopenia, Pregnancy, Newborn

## Abstract

**Background:**

Primary immune thrombocytopenia (ITP) can increase the risk of neonatal thrombocytopenia (NT). This study aimed to investigate the key factors for predicting the risk of NT.

**Methods:**

Data were retrospectively collected from all pregnant women with ITP from 2015 to 2021. Newborns were divided into two groups according to the presence or absence of NT. The parameters between the two groups were then compared. Next, the correlation between maternal platelet-to-lymphocyte ratio (PLR) and neonatal platelet count was analyzed by logistic regression and generalized additive model. Additionally, the relationships among the platelet counts of siblings were also determined.

**Results:**

A total of 147 maternal cases were included. NT was observed in 46 (31.72%) neonates. A history of previous children with NT appeared to have predictive value for NT (OR 16.484, 95% CI 2.212–122.858, *P* < 0.001), as the nadir gestational platelet (OR 0.958, 95% CI 0.93–0.988, *P* < 0.001). Correlation analysis of platelet count on postnatal day 1 and the nadir platelet count in 36 sibling neonates showed a positive correlation (*r*=0.684, *P*<0.001; *r*=0.900, *P*<0.05). PLR was divided into 3 groups via tertiles, and the incidence of NT was dramatically higher in the group with lower PLR during the second and third trimesters than in the other two groups (48.5% vs 33.3% vs 22%, *P*<0.05; 50% vs 21.3% vs 26.7%, *P*<0.001). Moreover, the risk of NT was markedly higher in the first trimester (PLR < 78.51; OR 0.975, 95% CI 0.951–0.999, *P*<0.05) and the second trimester (PLR < 20.41; OR, 0.899, 95% CI 0.820–0.985, *P*<0.05) compared to the third trimester.

**Conclusion:**

Our findings suggest that a history of previous children with NT is a significant factor for predicting NT in subsequent pregnancies. PLR in the first, second and third trimesters can also be used as a reference to predict NT risk.

## Introduction

Primary immune thrombocytopenia (ITP) is an acquired autoimmune thrombocytopenic disorder characterized by persistent or transient reduction in platelet counts. ITP is the most frequent cause of neonatal thrombocytopenia (NT) during early pregnancy. The incidence of ITP at pregnancy is 1/1000–10000, which accounts for 1%-4% of thrombocytopenias during pregnancy [1–4]. Platelet IgG-type autoantibody can pass through the placenta and result in NT, with a high risk of bleeding. Although major bleeding is rare, the incidence of intracranial hemorrhage (ICH) has been found in 0–1.8% of cases [5]. Hence, the prediction of platelet counts is critical for neonatal management. At present no clinical features or biomarkers in the mother can be used to accurately identify neonatal risk. Platelet-to-lymphocyte ratio (PLR) is an inflammatory biomarker that plays an important role in predicting the prognosis of patients with female reproductive system and gastrointestinal tumors [6–8]. PLR has also been used to predict the prognosis of other diseases [9–11]. However, to our knowledge, no studies have investigated the relationship between PLR and neonatal thrombocytopenia in offspring of women with ITP. Thus, we hypothesize that the maternal PLR, which is an indicator derived from platelets, lymphocytes and inflammatory response factor, may be associated with the risk of NT. In this study, a historical cohort study was conducted to analyze the neonates born to mothers with ITP in a single institution, and examine the factor associated with NT risk.

## Materials and methods

Ethics Committee of Peking University People's Hospital approved the study protocol (2020PHB396-01), and the need for informed consent was waived because it was a retrospective analysis of patient notes. In this retrospective study, pregnant women with ITP and their babies were recruited at a single institution from July 2015 to June 2021. Patients were included if they met the criteria of ITP during their pregnancy. Maternal ITP was defined as isolated thrombocytopenia (< 150×10^9^/L), with the exclusion of other causes of thrombocytopenia (e.g., hemolysis, elevated liver enzymes, low platelet count (HELP) syndrome, autoimmune diseases, sepsis, viral infections, and gestational thrombocytopenia) [12, 13]. Antiplatelet antibody detection was not necessarily performed in pregnant women and newborns.

A full blood count was performed on all newborns within the first 6 h of life. Thrombocytopenia was classified as mild (100–150×10^9^/L), moderate (50–100×10^9^/L), and severe (< 50×10^9^/L). To determine ICH (major bleeding), cranial USG was conducted on all neonates with severe thrombocytopenia. The minor sites of hemorrhage (umbilical and/or mucosal), baseline characteristics and clinical outcomes of pregnant women and newborns were analyzed.

Statistical tests were carried out with SPSS 24.0 software. The continuous variables with normal distribution were presented as mean ± standard deviation, whereas those with skewed distribution were expressed as median (quartile). Meanwhile, the categorical variables were shown as percentage. Univariate analysis of the two groups was conducted by Mann–Whitney and chi-square tests for continuous and categorical data, respectively. Spearman’s correlation coefficients were used to analyze the correlations. The factors associated with NT were identified by multivariate logistic regression analysis. The selection of covariates included in this study was based on our previous work and other relevant published studies.

Since platelet-to-lymphocyte ratio (PLR) is a continuous variable, its nonlinear correlation was analyzed using a generalized additive model (GAM). The inflection point was then measured for the nonlinear relationship. The correlation between the right and left sides of the inflection point was determined using a two-dimensional piecewise model. R package (http: www.R-project.org ) was used for data analysis. *P*<0.05 (two-sided) was deemed statistically significant.

## Results

**Fig. 1:**
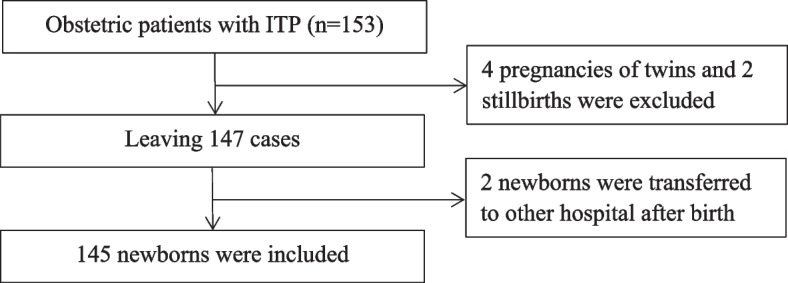
Flowchart for the selection of participants

During the study interval, 153 pregnancies with ITP were identified, excluding 4 pregnancies of twins and 2 stillbirths. A total of 147 pregnant women with ITP were ultimately included in this study (Fig. 1). The mean age of women at delivery was 30.7 ± 4.7 years. Of them, 128 (87.1%) women were known to have ITP based on thrombocytopenia plus a diagnosis of ITP before pregnancy, while the remaining 19 (12.9%) women were diagnosed with new-onset ITP during pregnancy. Among those with previously diagnosed disease, the average time elapsed from ITP diagnosis until pregnancy was 9.1 ± 6.6 years. Three (2.3%) patients had previously undergone splenectomy. Among those with new onset disease, thrombocytopenia occurred during the first (*n*=14, 73.7%), second (*n*=4, 21.1%), and third (*n*=1, 5.2%) trimesters.

Of the 147 pregnant women, 127 (86.4%) were treated for ITP during pregnancy, and the remaining 20 (13.6%) did not receive any treatment. The treatment options for 8 (5.5%), 44 (29.9%), 75 (51%) and 85 (57.8%) pregnant women were intravenous immunoglobulin (IVIG), corticosteroid, IVIG + corticosteroid and platelet transfusion at 24 h before or during delivery, respectively. The median maternal nadir platelet count was 22 × 10^9^/L during pregnancy and 18 × 10^9^/L before pregnancy. The median platelet count at delivery was 44 × 10^9^/L.

The median gestational week of newborns was 37.7 weeks. Of them, 27 (18.37%) newborns were premature. The median birth weight was 3139.4 g. Of them, 16 (10.9%) were low birth weight, and 2 (1.4%) were macrosomia. Two newborns were transferred to other hospital after birth with unknown platelet status. A total of 145 newborns were included in the analysis of platelet count.

NT was encountered in 46 (31.7%) pregnancies. Among thrombocytopenic newborns, 27 (58.7%), 14 (30.4%) and 5 (10.9%) had severe, moderate and mild thrombocytopenia, respectively. Thirteen (28.3%) neonates suffered from minor bleeding, while none of them had ICH. Since the underlying conditions and treatment status of 5 cases were unknown, only 41 newborns with thrombocytopenia were included. Of them, only 22 (53.6%) neonates received treatment: IVIG only (*n*=9), corticosteroids only (*n*=1), IVIG + corticosteroids (*n* = 6), IVIG + platelet transfusion (*n*=2), and IVIG + corticosteroids + platelet transfusion (*n* = 5). In contrast, the remaining 19 (46.4%) neonates did not receive any treatment, and their platelet counts increased spontaneously. The duration for the resolution of thrombocytopenia was 7–120 days.

Table 1 shows the comparison of the characteristics of neonates with and without thrombocytopenia. Platelet count was dramatically lower in neonates with thrombocytopenia compared to those without thrombocytopenia (92 × 10^9^/L vs 263 × 10^9^/L, respectively, *P* < 0.001) on postnatal day 1. There were no obvious differences in maternal age, ITP duration, mode of delivery, predelivery platelet count, and previously underwent splenectomy between the two groups. However, maternal nadir platelet counts ‘before pregnancy’ and ‘during pregnancy’ were lower in NT group than in non-NT group. Additionally, a history of previous children with NT was more commonly detected in NT group than in non-NT group (*P* = 0.005).

**Table 1:** Predictive parameters for neonatal thrombocytopenia

	Neonates with NT (*n* = 46)	Neonates without NT (*n* = 99)	*P* value
Maternal age at delivery (year)	30.33 ± 4.58	30.74 ± 4.75	0.625
Gestational age (weeks)	37.41 ± 1.72	37.83 ± 1.53	0.146
Cesarean	20 (43.5%)	58 (58.6%)	0.108
Primiparous	31 (67.4%)	68 (68.7%)	1
ITP diagnosed before pregnancy	42 (91.3%)	84 (84.8%)	0.428
Maternal nadir PC before pregnancy^a^ (× 10^9^/L)	14 (5.5, 27)	22 (8.75, 34.25)	0.011
Maternal nadir PC during pregnancy	16 (6.2, 8.5)	25 (13, 44.25)	0.003
First-trimester			
Maternal nadir PC^a^ (× 10^9^/L)	33.5 (17.25, 54)	54 (23, 75)	0.007
Lymphocyte count^a^ (× 10^9^/L)	1.89 (1.47, 2.37)	1.93 (1.5, 2.41)	0.833
PLR^a^	16.39 (7.55, 34.82)	25.53 (11.89, 53.23)	0.008
Second-trimester			
Maternal nadir PC^a^ (× 10^9^/L)	35.5 (17, 52.75)	44 (31, 72)	0.007
Lymphocyte count^a^ (× 10^9^/L)	1.97 (1.56, 2.33)	1.83 (1.49, 2.24)	0.833
PLR^a^	16.82 (7.45, 33.69)	21.86 (15.49, 45.91)	0.002
Third-trimester			
Maternal nadir PC^a^ (× 10^9^/L)	30.5 (24, 57)	54 (33, 92)	0.003
Lymphocyte count^a^ (× 10^9^/L)	1.98 (1.33, 2.35)	1.81 (1.4, 2.13)	0.136
PLR^a^	19.92 (9.13, 32.65)	28.14 (19.55, 49.53)	0.004
before delivery			
Last PC before delivery^a^ (× 10^9^/L)	42 (22.5, 75)	44.5 (29, 74)	0.595
Lymphocyte count^a^ (× 10^9^/L)	1.8 (1.4, 2.3)	1.7 (1.3, 2)	0.133
PLR^a^	36.63 (19.5, 61.36)	26.8 (13.17, 50.85)	0.078
Treatment during pregnancy			
IVIG only	3 (6.5%)	4 (4%)	0.679
Corticosteroids only	11 (23.9%)	33 (33.3%)	0.332
IVIG and corticosteroids	28 (60.9%)	47 (47.5%)	0.155
Other	30 (65.2%)	55 (55.6%)	0.284
Newborn			
Gender (male)	23 (50%)	59 (59.6)	0.287
Birth weight (gram)	3052.83 ± 486.75	3196.97 ± 427.22	0.073
Birth height (cm)	48.63 ± 2.52	49.52 ± 2.10	0.028
Low birth weight	7 (15.2%)	7 (7.1%)	0.138
Thrombocytopenia in siblings	7 (15.2%)	2 (2%)	0.005
PC at birth^a^ (× 10^9^/L)	92.5 (52, 138)	263 (216.75, 304.25)	0.000
Bleeding	15 (32.6%)	0 (0%)	0.000

^a^Median (P25, P75)

*ITP* Primary immune thrombocytopenia, *IVIG* Intravenous immunoglobulin, *NT* neonatal thrombocytopenia, *PC* Platelet counts, *PLR* Platelet-to-lymphocyte ratio

A multivariate logistic regression model was subsequently constructed. Maternal nadir platelet count ‘during pregnancy’ and a history of previous children with NT were identified as significant factors for predicting the risk of NT (OR 16.484, 95%CI 2.212–122.858; OR 0.958, 95%CI 0.93–0.988, respectively) (Table 2). However, there was no significant correlation between neonatal platelet count and maternal platelet count (Table 3).

**Table 2:** Multivariate analysis and presentation using adjusted models

Exposure	Adjusted model 1(OR, 95%CI, P)	Adjusted model 2(OR, 95%CI, P)
Previous children with NT	16.484 (2.212, 122.858) 0.006	18.241 (2.467, 134.847) 0.004
Maternal nadir PC ‘before pregnancy’	1.002 (0.976, 1.029) 0.879	1.001 (0.974, 1.029) 0.938
Maternal nadir PC ‘during pregnancy’	0.958 (0.93, 0.988) 0.006	0.958 (0.928, 0.990) 0.009

Adjusted model 1: adjusted for previous children with NT, maternal nadir PC ‘before pregnancy’ and ‘during pregnancy’. Adjusted model 2: adjusted for previous children with NT, maternal nadir PC ‘before pregnancy’ and ‘during pregnancy’, maternal age at delivery, primiparous, gestational age, splenectomy before pregnancy, diagnosis of ITP before pregnancy

*OR* Odds ratio, *CI* Confidence interval, *PC* Platelet counts

**Table 3:** Correlation analysis of platelets between newborns and mothers

		Maternal nadir PC ‘before pregnancy’	Maternal nadir PC ‘during pregnancy’	Last PC before delivery
Nadir PC of newborn	*r*	0.079	0.151	-0.11
	*P*	0.607	0.316	0.468
Neonatal PC at birth	*r*	0.192	0.211	0.068
	*P*	0.021	0.011	0.419

**Fig. 2:**
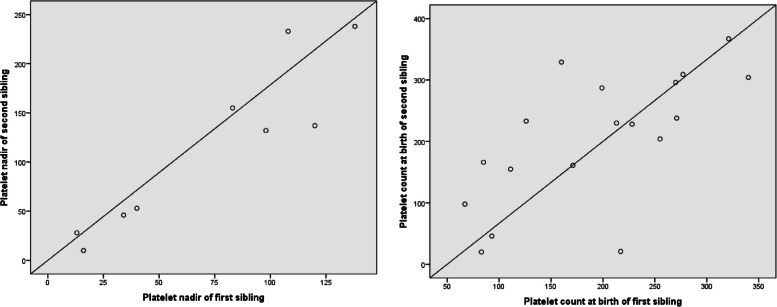
Correlations among the platelet counts of siblings (× 10.^9^/L)

Figure 2 shows the correlation between the first and second siblings. A total of nine groups of siblings were investigated, and a significant correlation was found between the platelet count postnatal day 1 and lowest platelet count (*r* = 0.684, *P* = 0.002; *r* = 0.900, *P* = 0.001, respectively).

Compared to newborns without thrombocytopenia, the mother of NT group had much lower levels of first-trimester platelet count (33.5 × 10^9^/L vs 54 × 10^9^/L, *P* = 0.007), first-trimester PLR (16.39 vs 25.53, *P* = 0.008), second-trimester platelet count (35.5 × 10^9^/L vs 44 × 10^9^/L, *P* = 0.007), second-trimester PLR (16.82 vs 21.86, *P* = 0.002), third-trimester platelet count (30.5 × 10^9^/L vs 54 × 10^9^/L, *P* = 0.003), and third-trimester PLR (19.92 vs 28.14, *P* = 0.004).

Based on the tertiles of PLR, there were no obvious differences in maternal age at delivery, neonatal birth weight, mode of delivery, and time of ITP diagnosed between the two groups. The occurrence of NT was remarkably higher in Q1 group than in Q3 group (Table 4). Through multiple comparison, there was a statistical difference between Q1 and Q3 groups, while there was no statistical difference between Q2 and Q1, Q3 during the first and second trimesters. In third trimester, there was a statistical difference between Q1 and Q2, Q3 groups, while there was no statistical difference between Q2 and Q3. Meanwhile, the platelet count of newborn on the 1st postnatal day was lower, especially during the second and third trimesters.

**Table 4:** Description of the baseline and clinical characteristics of mothers with primary immune thrombocytopenia

PLR, tertiles	Q1	Q2	Q3	*P* value
First trimester				
PLR	< 12.92	12.92~35.57	> 35.57	
N	33	32	32	
Maternal age at delivery (year)	30.42 ± 4.68	32.22 ± 5.44	31.59 ± 3.44	0.282
Gestational age (weeks)	36.85 ± 2.09	37.91 ± 1.28	38.34 ± 1.34	0.001
Birth weight (gram)	2998.79 ± 527.54	3121.25 ± 359.07	3268.44 ± 433.92	0.056
Cesarean	18 (54.5%)	15 (46.9%)	12 (37.5%)	0.386
ITP diagnosed before pregnancy	29 (87.8%)	31 (96.9%)	30 (93.8%)	0.362
NT	17 (51.5%)	11 (34.4%)	7 (21.9%)	0.044
Nadir PC of newborn^a^ (× 10^9^/L)	30 (13, 56)	53 (34, 83)	52 (16, 110)	0.205
PC at birth of newborn^a^ (× 10^9^/L)	197 (53, 256.5)	214 (133.25, 276.75)	257.5(170, 297)	0.026
Second trimester				
PLR	< 15.57	15.57~33.54	> 33.54	
N	41	42	43	
Maternal age at delivery (year)	30.78 ± 4.80	30.52 ± 5.11	31.07 ± 4.67	0.364
Gestational age (weeks)	36.88 ± 1.6	37.88 ± 1.53	38.21 ± 1.61	0.001
Birth weight (gram)	3032.93 ± 448.65	3098.33 ± 523.36	3230.93 ± 480.93	0.156
Cesarean	22 (53.7%)	25 (59.5%)	17 (39.5%)	0.166
ITP diagnosed before pregnancy	35 (85.4%)	36 (85.7%)	39 (90.7%)	0.711
NT	20 (48.8%)	14 (33.3%)	9 (20.9%)	0.027
Nadir PC of newborn^a^ (× 10^9^/L)	29 (10.75,52.75)	61.5 (43,86.75)	49 (15.5,81.5)	0.051
PC at birth of newborn^a^ (× 10^9^/L)	185 (68, 264)	213.5 (159.5, 298)	244 (162, 278)	0.025
Third trimester				
PLR	< 15.48	15.48~29.75	> 29.75	
N	46	47	47	
Maternal age at delivery (year)	30 ± 4.89	30.79 ± 5.19	31.62 ± 3.77	0.25
Gestational age (weeks)	36.85 ± 1.69	38 ± 1.22	38.34 ± 1.36	0.000
Birth weight (gram)	2966.3 ± 472.21	3215.11 ± 409.49	3152.71 ± 441.47	0.001
Cesarean	39 (84.8%)	41 (87.2%)	42 (89.4%)	0.804
ITP diagnosed before pregnancy	25 (54.3%)	28 (59.6%)	20 (42.6%)	0.239
NT	23 (50%)	10 (21.3%)	12 (25.5%)	0.006
Nadir PC of newborn^a^ (× 10^9^/L)	40 (19, 57)	54.5 (26.25, 86.75)	46.5 (19.5, 95.55)	0.26
PC at birth of newborn^a^ (× 10^9^/L)	172 (84.25, 279.25)	232(187, 280)	249(155, 283)	0.008

^a^Median (P25, P75)

*ITP* Primary immune thrombocytopenia, *NT* Neonatal thrombocytopenia, *PC* Platelet counts, *PLR* Platelet-to-lymphocyte ratio

Univariate logistic regression analysis demonstrated that PLR in Q1 (first trimester: OR 3.491, 95%CI 1.177–10.354, *P* = 0.024; second trimester: OR 3.386, 95%CI 1.296–8.845, *P* = 0.013; third trimester: OR 1.764, 95%CI 0.746–4.169, *P* = 0.196, respectively) was a risk factor for NT development. The PLR of Q1-Q3 was included as categorical variables in the logistic regression analysis. The same trend also was observed in the adjusted model. This trend was more notable in the second trimester ( OR 3.325, 95%CI 1.107–9.985, Ptrend = 0.032) (Table 5).

**Table 5:** Multivariate analysis and presentation using adjusted models

Exposure	Crude model (OR, 95%CI, P)	Adjusted model (OR, 95%CI, P)	P for trend
First-trimester PLR			
Q1	3.491 (1.177, 10.354) 0.024	3.375 (0.961, 11.848) 0.058	
Q2	1.721 (0.563, 5.260) 0.341	1.819 (0.573, 5.773) 0.310	
Q3	reference		0.058
Second-trimester PLR			
Q1	3.386 (1.296, 8.845) 0.013	3.325 (1.107 9.985) 0.032	
Q2	1.778 (0.668, 4.732) 0.249	2.056 (0.721, 5.866) 0.178	
Q3	reference		0.032
Third-trimester PLR			
Q1	2.750 (1.143, 6.616) 0.024	2.509 (0.78, 8.070) 0.123	
Q2	0.743 (0.284, 1.944) 0.545	0.661 (0.227, 1.925) 0.447	
Q3	reference		0.136

Adjusted model: previous children with NT, maternal age at delivery, primiparous, gestational age, splenectomy before pregnancy, diagnosis of ITP before pregnancy and peripheral blood lymphocyte count in the same trimester

*OR* Odds ratio, *CI* Confidence interval

**Fig. 3:**
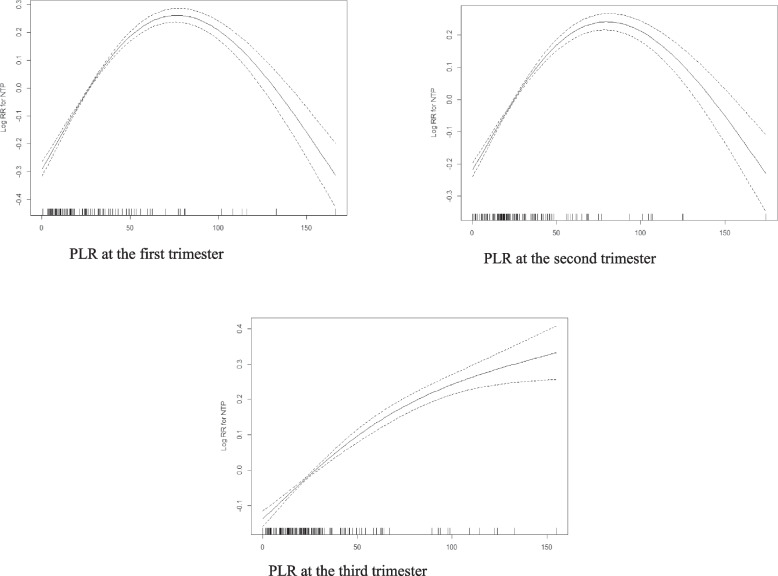
The nonlinearity of PLR and risk of NT occurrence

Furthermore, GAM and two-dimensional piecewise model were employed to clarify this nonlinear relationship and determine the potential threshold or saturation effect. The results showed a nonlinear relationship between maternal PLR and NT risk (Fig. 3). Notably, the inflection points were 78.15, 20.41 and 21.58 in the first, second and third trimester, respectively. Through the use of two-dimensional piecewise logistic regression model, different OR trends were detected on the left and right sides of the inflection point (Table 6).

**Table 6:** Nonlinear analysis via the two-dimensional piecewise model

	Inflection point of PLR	*P* value	*OR*	95%*CI*
PLR at the first trimester	< 78.51	0.045	0.975	0.951–0.999
	≥ 78.51	0.833	0.991	0.908–1.081
PLR at the second trimester	< 20.41	0.022	0.899	0.82–0.985
	≥ 20.41	0.626	0.995	0.977–1.014
PLR at the third trimester	< 21.58	0.13	0.939	0.866–1.019
	≥ 21.58	0.362	0.991	0.972–1.011

*PLR* Platelet-to-lymphocyte ratio

## Discussion

Due to the fact that antiplatelet antibodies can pass through the placenta and lead to thrombocytopenia, the neonates born to mothers with ITP are at risk of NT and bleeding. Thus, managing maternal ITP is of utmost importance to reduce the risk of NT [14, 15]. Platelet counts of newborns may fall during the first 2 to 5 days after delivery, which may be attributed to the maturation of the spleen and other clearance mechanisms [16, 17]. In our study, the platelet count of newborns fell to a nadir on postnatal day 4.

Although remarkable progress has been made in the treatment of maternal ITP, the incidence of NT remains high. In a previous study 15.4–67.0% of babies born to mothers with ITP had thrombocytopenia, and the ratio of severe thrombocytopenia was reported as 8.0–29.9% among these neonates [16]. A total of 46 (31.7%) pregnancies encountered NT were identified in our study. Despite that prior investigations have identified several risk factors associated with the development of NT, the association between several maternal factors and neonatal platelet count is still contradictory. A previous study has shown an inverse association among maternal platelets, antibodies and timing of diagnosis [5]. Maternal treatment for ITP, such as splenectomy, is not significantly related to the risk of neonatal thrombocytopenia [13]. Similarly, maternal response to treatment does not confer protection against thrombocytopenia development among the newborns. On the other hand, there is no robust evidence to suggest that neonates from ITP women with poor response or refractory to treatment have a higher risk of severe thrombocytopenia [17]. Numerous studies have reported that a history of previous children with thrombocytopenia is the most important parameter to predict the risk of NT [13, 16, 18–20]. Consistent with these studies, we found that a history of previous children with NT exhibited good prognostic value for predicting NT in subsequent pregnancies (OR 16.484, 95%CI 2.212–122.858). The multivariate logistic regression analysis also revealed that a low maternal platelet count during pregnancy was associated with NT risk. Nevertheless, in the correlation analysis, no significant correlation was found between maternal and neonatal platelet counts. Considering the severe impact of NT, early disease prediction could potentially reduce maternal anxiety and assist in guiding therapy.

ITP is classified as an immune-mediated disorder. It has been recently reported that multifactorial autoimmune mechanisms, such as faulty CD4 + Tregs, are involved in the pathogenesis of ITP [21]. Lymphocytopenia is a common feature of many chronic autoimmune diseases. Deel et al. [22] demonstrated that progression to lower lymphocyte counts within the first few months is a significant predictive factor for chronic ITP. Song et al. [23] suggested that PLR is a valuable parameter for assessing the risk of ITP recurrence. PLR has initially been used as a systemic inflammatory biomarker for predicting the prognosis of neoplastic disorders [24–26]. More recently, PLR has been employed as a prognostic marker in a variety of cardiovascular diseases [27]. PLR is a comprehensive reflection of two pivotal inflammatory pathways, which can easily be measured from a full blood count, and it is more accurate than the lymphocyte or platelet count alone. Hence, there is no way to predict NT based on maternal platelet count [28, 29], but PLR may have an additive role in predicting NT.

This retrospective cohort study evaluated the relationship between PLR at pregnancy and NT occurrence. The results showed that PLR < 78.51 at the first trimester and PLR < 20.41 at the second trimester were associated with NT risk. However, when the PLR values were > 78.51 and > 20.41 at the first and second trimesters, respectively, the risk of NT was basically the same. Therefore, PLR can serve as a marker for predicting NT occurence. The nonlinear regression analysis of PLR also supported its better clinical utility. The results demonstrated that an increase in PLR was significantly associated with the decreased risk of NT, implying that the clinicians can directly assess the risk of NT based on PLR.

Our study has some strengths. Firstly, we introduced PLR into the risk assessment model for NT occurrence, and included different stages of pregnancy. Secondly, PLR was used as a continuous variable as well as a categorical variable, which is useful in evaluating the robustness of data analysis. Thirdly, a logistic regression model was constructed to evaluate the linearity, while GAM was used to explain the nonlinearity. GAM is powerful tool that can be used to determine the actual relationship between PLR and NT risk. However, due to its retrospective nature, our study has several limitations. The patients in this study were predominantly recruited from a tertiary care center, and most of them were followed up by only one of the authors. Hence, the generalizability of the results of this study may be limited due to potential referral bias. Moreover, we did not compare the effectiveness of PLR at the three trimesters in predicting NT risk. Additionally, because PLR is based on the platelet and lymphocyte count, the comparison of performance among PLR, platelet count, and lymphocyte counts should be performed. However, the maternal platelet count can be interfered with by treatment which including specific types of drugs, doses, and treatment start times. Therefore, larger studies are warranted to verify our findings.

## Conclusion

In summary, newborns with maternal ITP have a high susceptibility to NT. A history of previous children with NT is a significant factor for predicting NT in subsequent pregnancies. PLR in the first, second and third trimesters can also be used as a reference to predict the occurrence of NT.

## Data Availability

The data are not publicly available due to the hospital policy and personal privacy, but are available from the corresponding author on reasonable request.
